# Base Station Selection for Hybrid TDOA/RTT/DOA Positioning in Mixed LOS/NLOS Environment

**DOI:** 10.3390/s20154132

**Published:** 2020-07-24

**Authors:** Zhongliang Deng, Hanhua Wang, Xinyu Zheng, Lu Yin

**Affiliations:** School of Electronic Engineering, Beijing University of Posts and Telecommunications, Beijing 100876, China; dengzhl@bupt.edu.cn (Z.D.); buptzxy@bupt.edu.cn (X.Z.); inlu_mail@bupt.edu.cn (L.Y.)

**Keywords:** hybrid positioning, base station selection, geometric dilution of precision, 5G system

## Abstract

The fifth generation (5G) cellular communication system is designed to support Time Difference of Arrival (TDOA), Round-Trip Time (RTT), and Direction of Arrival (DOA) measurements for indoor positioning. To mitigate the positioning error caused by non-line-of-sight (NLOS), existing base station selection methods identify channel conditions and only use line-of-sight (LOS) signals for positioning. However, different selected base station combination would lead to a different geometric dilution of precision (GDOP), base station selection based only on channel condition is not fully applicable for the hybrid positioning. This paper derives the GDOP for the hybrid TDOA, RTT, and DOA positioning, and proposes a GDOP-assisted base station selection method, which is based on both channel conditions and GDOP value changes. The simulation shows that using the proposed base station selection method could lead to higher positioning accuracy than base station selection based only on channel condition. In the simulation, in the side region of the scenario, where the change of selected base station combination causes a notable increment in GDOP value, the positioning accuracy improvement caused by the proposed method is greater than that in the center region.

## 1. Introduction

Indoor positioning plays an important role in the location-based service, the Internet of Things (IoT), and health care [1,2]. As one of the most widely used wireless systems, cellular systems has supported user equipment (UE) positioning since the second generation (2G) [3]. In order to meet the growing location service market, the 3rd Generation Partnership Project (3GPP) sets higher requirements to indoor positioning accuracy for the fifth generation (5G) cellular communication system [4,5]. The Long Range Radio (LoRa), Wi-Fi, Bluetooth, and other wireless networks for IoT scenarios also have positioning function, and have the advantages of low cost, low power consumption and low complexity [6,7]. However, their limited bandwidth, power consumption, and complexity result in a low positioning accuracy. Unlike them, the 5G system can support higher cost, power consumption, and complexity. The 5G system uses technical features including ultra-dense network (UDN), millimeter wave (mm-wave), massive multiple-input-multiple-output (MIMO) and 400 MHz bandwidth to achieve high-accuracy indoor positioning [8]. The 5G system can also support multiple types of positioning measurements, such as Round-Trip Time (RTT), Direction of Arrival (DOA), and Time Difference of Arrival (TDOA) [9]. To measure RTT and DOA, base station (BS) need to receive uplink signals from UE [8]. Due to the limited uplink signal power, these two measurements are only available to the serving BS. TDOA measurement is obtained by the Observed Time Difference of Arrival (OTDOA) technology, which enables BSs send positioning reference signal (PRS) and UE measures the difference of PRSs’ arrival time [10]. These measurements enable the 5G system to support hybrid multi-measurements positioning. Existing studies already demonstrated that positioning system could achieve a lower Cramer–Rao Low Bounds (CRLB) of values of positioning accuracies as the number of positioning measurements increases [11]. Therefore, the support for hybrid positioning makes the 5G system have great potential in positioning performance.

The major error sources of positioning measurement in wireless positioning include system noise and propagation error [12]. The system noise is the estimation error generated in signal processing, and the propagation error is caused by wireless signal reflection and scattering during propagation. For non-line-of-sight (NLOS) conditions, the condition can be subdivided into two conditions. The one is the condition where direct path signal is partially obstructed but could still be received, the other one is the condition where the direct path signal is completely obstructed and only the signals from scatterers could be received [13]. The large bandwidth makes the high sampling rate and shortens the sampling time interval of the 5G system, so that the wireless channel has better resolution in time. The time delay of most multipath components in the channel is longer than the sampling interval, which makes it is easier to estimate them in the time domain [14]. As a result, for the 5G system, it is easy to identify the signal paths. In partially obstructed NLOS condition, the propagation error could be avoided by identify the direct path, but the system noise is higher than LOS condition due to the lower range of values of signal to noise ratio (SNR). In completely obstructed NLOS condition, since the system would mistake reflected path signal for direct path signal, the reflection will introduce a large propagation error. Fortunately, in the indoor environment, due to UDN technology, 5G BSs will have extremely high density, as a result, the closer distance between UE and the BSs will reduce the occurrence of completely blocked NLOS [15]. However, the partially blocked NLOS condition caused by moving objects is still a problem to solve.

Identifying the LOS/NLOS signals and selecting LOS BSs to estimate position has become a major approach to mitigate the effect of NLOS caused measurement errors on positioning results inside and outside buildings. Existing LOS/NLOS condition-based BS selection methods can be divided into coherence criterion-based methods and channel characteristics-based methods. In coherence criterion-based BS selection method, the criterion is the residual between the positioning measurement and position result estimated by the measurements is used as an indicator of the coherence to select BSs [16]. The base stations with the smallest residuals are selected for final positioning. The results show that the BS in the NLOS condition is more likely to have a larger residual [17,18]. But when there is not enough LOS BSs to provide a high-accuracy initial position, this method cannot provide reliable and accurate BS selection. In channel characteristics-based BS selection method, the receiver receives and measures the channel parameters of reference signal, then compares them with the prior statistical characteristics of channel parameters in different conditions to estimate whether the channel is under LOS or NLOS conditions [19]. The variances of signal arrival angle, delay and strength are quite different in LOS and NLOS conditions. The prior probability statistics of channel parameters can be used to calculate probability of whether BS is in NLOS condition and the probability of detection can be up to 95% [20]. However, in hybrid TDOA, RTT, DOA positioning, if serving BS is in NLOS condition, the LOS/NLOS condition-based selection would not select it for positioning, which will have a great impact on the positioning capability. Therefore, it is necessary to re-evaluate whether the BS selection based only on LOS/NLOS condition is suitable for the hybrid positioning.

Not only the measurement error of positioning signals, but also the layout of the positioning BSs and number of measurements have great effect on positioning error [21]. The geometric dilution of precision (GDOP) is widely used in satellite positioning to express the positioning errors caused by satellite layout and number of available satellites, it is defined as the ratio of positioning accuracy limit to positioning signal measurement accuracy [22]. Existing researches have proved that when the sources are in different layout and different number, the positioning accuracy and GDOP value would change. Therefore, changes in GDOP should also be considered for the BS selection. GDOP for pseudorange-based positioning has been widely used in the selection of satellites, a group of satellites with the smallest GDOP value has the potential to achieve better performance [23,24]. However, the GDOP for the hybrid TDOA, RTT, and DOA positioning, and the BS selection methods considering GDOP, need to be studied.

In this paper, we propose a GDOP-assisted BS selection method in LOS and NLOS mixed environment. We first derive the GDOP for the hybrid TDOA, RTT, and DOA positioning. Using the derived GDOP, the proposed BS selection method is an enhancement on LOS/NLOS condition-based selection, not only a channel condition, the GDOP changes are also considered. The LOS BSs and the NLOS BSs, that have a better GDOP, would be selected for positioning. The simulation shows that the proposed method can lead to a higher positioning accuracy than the selection based only on LOS/NLOS condition.

This paper is organized as follows: Section 2 describes the system model of the hybrid TDOA, RTT and DOA positioning in indoor 5G system. In Section 3, the GDOP for the three types of measurements-based hybrid positioning are derived. Section 4 describes the proposed GDOP-assisted BS selection method. Simulation results and analysis are presented in Section 5. Finally, we draw our conclusions in Section 6. A list of symbols that are used in the paper is given in Table 1.

## 2. System Model

**Figure 1:**
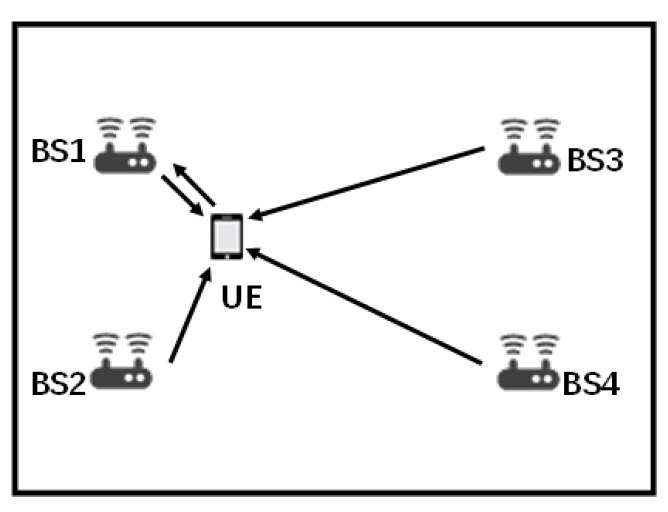
The system model of fifth generation (5G) system indoor positioning, base station being denoted by “BS” and user equipment being denoted by “UE”.

The system model of 5G cellular system based indoor positioning is shown in Figure 1. We consider the UE could receive downlink PRS from *M* BSs and obtain TDOAs with the number of *M* minus 1. For the model shown in Figure 1, *M* is equal to 4. The serving BS is denoted as BS 1, and the rest BSs are sorted by the distance to UE, the farthest is numbered M. Only the serving BS could receive uplink signal from UE and obtain RTT and DOA.

**Figure 2:**
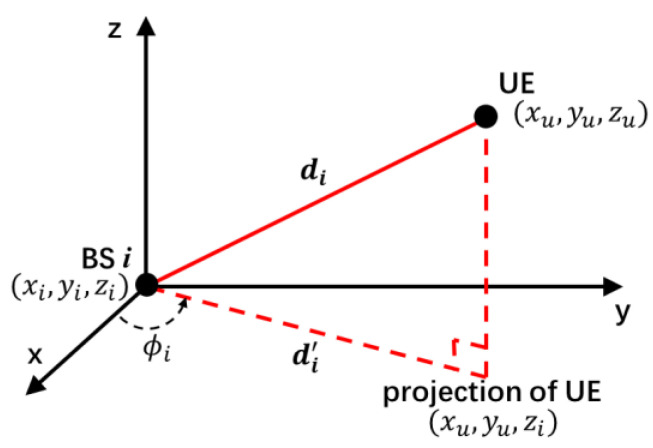
The three-dimension Cartesian coordinate for US positioning.

In the following description, the position of UE is denoted by $u={\left(x_{u},y_{u},z_{u}\right)}^{T}$ and BS positions are denoted by $bs_{i}={\left(x_{i},y_{i},z_{i}\right)}^{T},\;\;i=1,\;2,\;3,\ldots ,\;M$. Figure 2 shows the three-dimension Cartesian coordinate of system.

The real distance from UE to BS i is expressed as

(1) $$\begin{array}{c} d_{i}=\|bs_{i}-u\|=\sqrt{{\left(x_{i}-x_{u}\right)}^{2}+{\left(y_{i}-y_{u}\right)}^{2}+{\left(z_{i}-z_{u}\right)}^{2}},\\ i=1,2,3,\ldots ,M. \end{array}$$

The real distance from projection of UE to BS i is expressed as

(2) $$\begin{array}{c} d_{1}^{'}=\|bs_{i}-{\left(x_{u},y_{u},z_{i}\right)}^{T}\|=\sqrt{{\left(x_{i}-x_{u}\right)}^{2}+{\left(y_{i}-y_{u}\right)}^{2}},\\ i=1,2,3,\ldots ,M. \end{array}$$

The RTT measurement result between BS 1 and UE is expressed as

(3) $$r_{t}=ct_{rtt}=d_{1}+e_{t},$$

where $r_{t}$ is the ranging result, $t_{rtt}$ is the measured round-trip time, and c is the speed of light, $d_{1}$ is the real distance and $e_{t}$ is the measurement error.

The TDOA ranging result is expressed as

(4) $$r_{i,1}=c\left(t_{1}-t_{i}\right)=d_{1}-d_{i}+e_{i,1}=d_{i,1}+e_{i},\;\;i=2,3,4,\ldots ,M,$$

where $r_{i,1}$ is the ranging difference calculated by TDOA measurement, $t_{1}$ is measured PRS arrival time from BS 1 to UE, and $t_{i}$ is measured PRS arrival time from BS *i* to UE, and c is the speed of light, $d_{i,1}$ is the difference between real distances and $e_{i}$ is the measurement error.

The DOA measurement from UE to BS 1 is expressed as

(5) $$a_{a}=\phi_{1}+e_{a}=\tan^{-1}\frac{y-y_{1}}{x-x_{1}}+e_{a},$$

where $a_{a}$ is the measured azimuth angle, $\phi_{1}$ is the real azimuth angle and $e_{a}$ is the measurement error.

## 3. GDOP for The Hybrid TDOA/RTT/DOA Positioning

GDOP is defined as the ratio of the accuracy limitation of a position fix to the accuracy of measurements [22]. The GDOP value is a function of the position of BSs and UE, and can be used to analyze the influence of the geometric relationship between BSs and UE on the positioning capability. To calculate the GDOP value of different selected BS combinations, it is intuitive to compare and analyze the positioning capability changes caused by the different positions of the selected BSs.

GDOP for the hybrid TDOA, RTT, and DOA positioning is derived in the following. In the indoor environment, the antennas of BSs are usually deployed under the ceiling and have same height, which lead to a high vertical dilution of precision and makes the system cannot provide reliable vertical positioning results [25]. Therefore, the vertical positioning results are usually provided by other sensors [26]. Therefore, only the horizontal positioning is considered in this paper. Using $\left(x^{\prime },y^{\prime }\right)$ to express the position estimation result and $f\left(x^{\prime },y^{\prime }\right)$ to express the functional relationship of estimated position and measurement errors. For DOA measurement error, $f\left(x^{\prime },y^{\prime }\right)$ is rewritten as $f_{a}\left(x^{\prime },y^{\prime }\right)$. For RTT measurement error, $\;f\left(x^{\prime },y^{\prime }\right)$ is rewritten as $f_{t}\left(x^{\prime },y^{\prime }\right)$. For RTT measurement error, $\;f\left(x^{\prime },y^{\prime }\right)$ is rewritten as $f_{i,1}\left(x^{\prime },y^{\prime }\right)$,  i=2,3,4,…,M. In the system containing *M* BSs, the DOA measurement equations can be written as

(6) $$e_{a}=f_{a}\left(x^{\prime },y^{\prime }\right)=a_{a}-\tan^{-1}\frac{y^{\prime }-y_{1}}{x^{\prime }-x_{1}}.$$

The RTT measurement equations can be written as

(7) $$e_{t}=f_{t}\left(x^{\prime },y^{\prime }\right)=r_{t}-\sqrt{{\left(x_{1}-x^{\prime }\right)}^{2}+{\left(y_{1}-y^{\prime }\right)}^{2}+{\left(z_{1}-z_{u}\right)}^{2}},$$

where the value of $z_{u}$ is known. 

The TDOA measurement equations can be written as

(8) $$\begin{array}{c} e_{i}=f_{i,1}\left(x^{\prime },y^{\prime }\right)=r_{i,1}-\sqrt{{\left(x_{1}-x^{\prime }\right)}^{2}+{\left(y_{1}-y^{\prime }\right)}^{2}+{\left(z_{1}-z_{u}\right)}^{2}}+\\ \sqrt{{\left(x_{i}-x^{\prime }\right)}^{2}+{\left(y_{i}-y^{\prime }\right)}^{2}+{\left(z_{i}-z_{u}\right)}^{2}},\;i=2,3,4,\ldots ,M. \end{array}$$

To achieve the GDOP for hybrid TDOA, RTT and DOA positioning, we first need to linearize the measurement equations. Using the first order Taylor series to approximate $f\left(x^{\prime },y^{\prime }\right)$ when UE locates at $\left(x_{p},y_{p}\right)$, it is obtained as

(9) $$f\left(x^{\prime },y^{\prime }\right)\approx f\left(x_{p},y_{p}\right)+\left(x^{\prime }-x_{p}\right)\frac{\partial f\left(x_{p},y_{p}\right)}{\partial x}+\left(y^{\prime }-y_{p}\right)\frac{\partial f\left(x_{p},y_{p}\right)}{\partial y}.$$

When there are no measurement errors, the position estimation result $\left(x^{\prime },y^{\prime }\right)$ is equal to UE’s real location $\left(x^{0},y^{0}\right)$. Equation (9) is rewritten as

(10) $$0=f\left(x_{p},y_{p}\right)+\left(x^{0}-x_{p}\right)\frac{\partial f\left(x_{p},y_{p}\right)}{\partial x}+\left(y^{0}-y_{p}\right)\frac{\partial f\left(x_{p},y_{p}\right)}{\partial y}.$$

When the measurement errors are not equal to zero and use e to express all types of the measurement errors, $e_{t}$, $e_{a}$, and $e_{i}$, Equation (9) is rewritten as

(11) $$e=f\left(x_{p},y_{p}\right)+\left(x^{\prime }-x_{p}\right)\frac{\partial f\left(x_{p},y_{p}\right)}{\partial x}+\left(y^{\prime }-y_{p}\right)\frac{\partial f\left(x_{p},y_{p}\right)}{\partial y}.$$

Let position estimation error vector $∆u={\left(e_{x},e_{y}\right)}^{T}$, where $e_{x}=x\prime -x^{0}$, $e_{y}=y\prime -y^{0}$. Subtracting Equation (11) from Equation (10), the equation of e and ∆u can be obtained as:

(12) $$e=e_{x}\frac{\partial f\left(x_{p},y_{p}\right)}{\partial x}+e_{y}\frac{\partial f\left(x_{p},y_{p}\right)}{\partial y}.$$

For RTT measurement error $e_{t}$, Equation (12) is rewritten as

(13) $$\begin{array}{c} e_{t}=e_{x}\frac{\partial f_{t}\left(x_{p},y_{p}\right)}{\partial x}+e_{y}\frac{\partial f_{t}\left(x_{p},y_{p}\right)}{\partial y}\\ =e_{x}\frac{x_{1}-x_{p}}{\sqrt{{\left(x_{1}-x_{p}\right)}^{2}+{\left(y_{1}-y_{p}\right)}^{2}+{\left(z_{1}-z_{p}\right)}^{2}}}+e_{y}\frac{y_{1}-y_{p}}{\sqrt{{\left(x_{1}-x_{p}\right)}^{2}+{\left(y_{1}-y_{p}\right)}^{2}+{\left(z_{1}-z_{p}\right)}^{2}}}. \end{array}$$

For DOA measurement error $e_{a}$, Equation (12) is rewritten as

(14) $$\begin{array}{l} e_{a}=e_{x}\frac{\partial f_{a}\left(x_{p},y_{p}\right)}{\partial x}+e_{y}\frac{\partial f_{a}\left(x_{p},y_{p}\right)}{\partial y}\\ \;=e_{x}\frac{y_{p}-y_{1}}{{\left(x_{1}-x_{p}\right)}^{2}+{\left(y_{1}-y_{p}\right)}^{2}}+e_{y}\frac{x_{1}-x_{p}}{{\left(x_{1}-x_{p}\right)}^{2}+{\left(y_{1}-y_{p}\right)}^{2}}. \end{array}$$

For TDOA measurement errors $e_{i,1}$, i=2,3,…,M, Equation (12) is rewritten as

(15) $$\begin{array}{c} e_{i}=e_{x}\frac{\partial f_{i,1}\left(x_{p},y_{p}\right)}{\partial x}+e_{y}\frac{\partial f_{i,1}\left(x_{p},y_{p}\right)}{\partial y}\\ =e_{x}\left(\frac{x_{i}-x_{p}}{\sqrt{{\left(x_{i}-x_{p}\right)}^{2}+{\left(y_{i}-y_{p}\right)}^{2}+{\left(z_{i}-z_{p}\right)}^{2}}}-\frac{x_{1}-x_{p}}{\sqrt{{\left(x_{1}-x_{p}\right)}^{2}+{\left(y_{1}-y_{p}\right)}^{2}+{\left(z_{1}-z_{p}\right)}^{2}}}\right)\\ +e_{y}\left(\frac{y_{i}-y_{p}}{\sqrt{{\left(x_{i}-x_{p}\right)}^{2}+{\left(y_{i}-y_{p}\right)}^{2}+{\left(z_{i}-z_{p}\right)}^{2}}}-\frac{y_{1}-y_{p}}{\sqrt{{\left(x_{1}-x_{p}\right)}^{2}+{\left(y_{1}-y_{p}\right)}^{2}+{\left(z_{1}-z_{p}\right)}^{2}}}\right). \end{array}$$

After linearization, measurement equations in Equations (6)–(8) can be formulated as the following matrix form:

(16) $$A∆u=\vec{e}$$

where

(17) $$A=\left[\begin{array}{cc} \alpha_{a} & \beta_{a}\\ \alpha_{t} & \beta_{t}\\ \alpha_{2} & \beta_{2}\\ \alpha_{3} & \beta_{3}\\ \vdots & \vdots \\ \alpha_{M} & \beta_{M} \end{array}\right],\;∆u=\left[\begin{array}{c} e_{x}\\ e_{y} \end{array}\right],\;\vec{e}=\left[\begin{array}{c} e_{a}\\ e_{t}\\ e_{2}\\ \begin{array}{c} e_{3}\\ \vdots \\ e_{M} \end{array} \end{array}\right],\;\alpha =\frac{\partial f\left(x_{p},y_{p}\right)}{\partial x},\;\beta =\frac{\partial f\left(x_{p},y_{p}\right)}{\partial y}.$$

In the cellular communication system, the three different type measurements, TDOA, RTT and DOA are measured independently by different reference signals and the errors of those different type measurements can be assumed as mutually independent differently zero-mean Gaussian distribution [27,28]. For different TDOA measurements, measurement errors $e_{i,1}$, i=2,3,…,M can be assumed to be independent and identically distributed [29]. 

For all elements in the error vector $\vec{e}$,

(18) $$E\left(e_{i}\right)=0,i=a,e,t,2,3,\ldots ,M\;Cov\left(e_{i}e_{j}\right)=0,i\neq j\;and\;i,\;j=a,e,t,2,3,\ldots ,M.$$

Standard deviations of different kind measurement error have the following statistical properties:

(19) $$\begin{array}{l} Var\left(e_{i}\right)=\sigma_{tdoa}^{2},i=2,3,\ldots ,M\\ Var\left(e_{t}\right)=\sigma_{t}^{2}=k_{t}{}^{2}\sigma_{tdoa}^{2}\\ Var\left(e_{a}\right)=\sigma_{a}^{2}=k_{a}{}^{2}\sigma_{tdoa}^{2}, \end{array}$$

where $\sigma_{tdoa}^{2}$ is the variance of TDOA measurement error, and $\sigma_{t}^{2}$ is the variance of RTT measurement error, and the $\sigma_{a}^{2}$ is the DOA measurement error.

According to the weighted least square (WLS), the solution to Equation (16) is given by

(20) $$∆u={\left(A^{T}WA\right)}^{-1}A^{T}W\vec{e}.$$

where W is the weighted matrix, which can be obtained by calculating the covariance of the measurement errors as

(21) $$Cov\left(\vec{e}\right)=E\left(\left[\begin{array}{c} e_{a}\\ e_{t}\\ e_{2}\\ \begin{array}{c} e_{3}\\ \vdots \\ e_{M} \end{array} \end{array}\right]\left[\begin{array}{cccccc} e_{a} & e_{t} & e_{2} & e_{3} & \ldots & e_{M} \end{array}\right]\right)=\sigma_{tdoa}^{2}\left[\begin{array}{cccccc} k_{a}{}^{2} & 0 & 0 & 0 & \ldots & 0\\ 0 & k_{t}{}^{2} & 0 & 0 & \ldots & 0\\ 0 & 0 & 1 & 0 & \ldots & 0\\ 0 & 0 & 0 & 1 & \ldots & 0\\ \vdots & \vdots & \vdots & \vdots & \ddots & \vdots \\ 0 & 0 & 0 & 0 & \ldots & 1 \end{array}\right]=\sigma_{tdoa}^{2}\Sigma$$

(22) $$W=\Sigma^{-1}$$

Since $E\left(e_{i}\right)=0,i=a,e,t,2,3,\ldots ,M$, the mean of ∆u can be calculated by

(23) $$E\left(∆u\right)=E\left({\left(A^{T}WA\right)}^{-1}A^{T}W\vec{e}\right)={\left(A^{T}WA\right)}^{-1}A^{T}WE\left(\vec{e}\right)=0.$$

The covariance matrix of estimated position error ∆u can be expressed as

(24) $$\begin{array}{c} Cov\left(∆u\right)=E\left(∆u∆u^{T}\right)=E\left({\left(A^{T}{\left(\vec{e}{\vec{e}}^{T}\right)}^{-1}A\right)}^{-1}\right)={\left(A^{T}Cov{\left(\vec{e}\right)}^{-1}A\right)}^{-1}=\\ {\left(A^{T}\frac{1}{\sigma_{tdoa}^{2}}WA\right)}^{-1}=\sigma_{tdoa}^{2}{\left(A^{T}WA\right)}^{-1}. \end{array}$$

Therefore, according to the definitions, GDOP is defined as the ratio of positioning error to positioning signal measurement error [22]. For the independent non-identical measurement errors, the GDOP calculated using WLS is called weighted GDOP [30,31] and the GDOP of the hybrid TDOA, RTT and DOA positioning is given by the trace of the inverse of the $A^{T}WA$ matrix

(25) $$\mathrm{GDOP}=\sqrt{\frac{Var\left(e_{x}\right)+Var\left(e_{y}\right)}{\sigma_{tdoa}^{2}}}=\sqrt{\frac{tr\left(Cov\left(∆u\right)\right)}{\sigma_{tdoa}^{2}}}=\sqrt{tr\left({\left(A^{T}WA\right)}^{-1}\right)}.$$

## 4. GDOP-Assisted Base Station Selection Method

Utilizing the GDOP derived in the above section, a BS selection method based on channel condition and GDOP changes is proposed for the hybrid positioning in this section. The proposed method is used for selecting BSs and corresponding measurements to positioning after UE has already measured RTT, DOA and TDOAs. The selection procedure consists of the following four steps:

1. Identify the channel condition of BSs and classify LOS BSs as the selected BSs.
2. Estimate an initial position result.
3. Find the unselected BS which could reduce GDOP most.
4. Determine whether to mark the BS found in the step 3 as selected. If yes, jump back to step 2.

**Figure 3:**
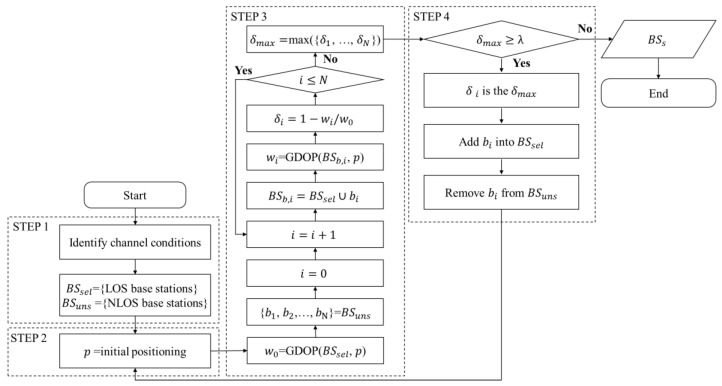
The flowchart of geometric dilution of precision (GDOP)-assisted BS selection method.

The procedure is shown as the flowchart in Figure 3 and the detailed description of steps follows.

In the step 1, a channel characteristics-based LOS/NLOS condition identification method, as proposed in [20], is used for identifying the channel conditions of each BS. After the identification, the BSs in LOS condition are classified into $BS_{sel}$ as the selected BS combination and the NLOS BSs are classified $BS_{uns}$ as the unselected BS combination. This step is identical to the BS selection method based only on LOS/NLOS condition [20].

In the step 2, an initial position, p, is estimated. If $BS_{sel}$ has enough BSs to estimate UE position, the p is estimated by measurements from selected BS combination, and result is same as the positioning based on the LOS/NLOS condition-based BSs selection. Otherwise, if $BS_{sel}$ does not have enough BSs, the initial position is estimated by all measurements, and result is same as that without any BSs selection.

In the step 3, the GDOP decrement rate caused by each element in $BS_{uns}$ is calculated and the BS that reduce GDOP most is found. At first, the GDOP at p for selected BSs is calculated and denoted by $w_{0}$. Then, for each BS in $BS_{uns},$ which is denoted by $b_{i}$, calculate the new GDOP at p for the union of $BS_{sel}$ and $b_{i}$, and denote the GDOP as $w_{i}$. The lower the GDOP value, the higher the positioning capability. Therefore, the GDOP decrement rate, calculated as $\delta_{i}=1-w_{i}/w_{0}$, could express the improvement of positioning capability. Finally, find the unselected BS which reduce the GDOP value most.

In the step 4, determine whether to add the BS, which reduce GDOP value most, into $BS_{sel}$ and continue selection. A threshold, λ, which is used to judge whether to add the BS found in step 3, needs to be calculated using the statistical learning method before BS selection procedure. If the largest GDOP decrement rate in step 3 is higher than the threshold, the corresponding unselected BS would be selected and remove from $BS_{uns}$. If the largest GDOP decrement rate is lower than the threshold, the selection procedure is finish and the $BS_{sel}$ is the selected BSs.

**Figure 4:**
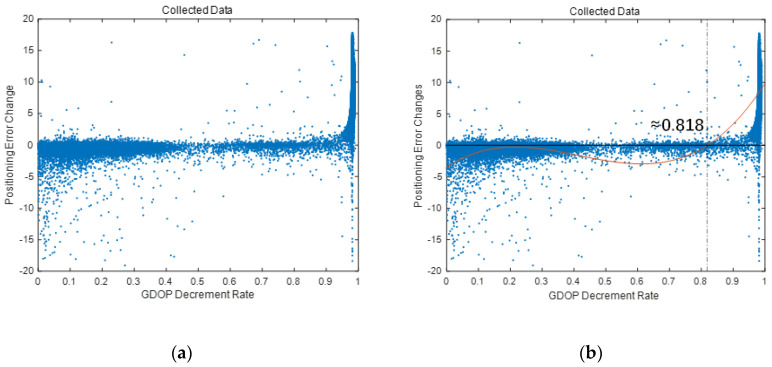
The distribution of collected GDOP decrement rates and positioning error changes in simulation. (**a**) the distribution of collected data. (**b**) the linear regression result.

To find the threshold λ, firstly, positioning measurements that UE received at test points on different random location and different time, different channel conditions of BSs, need to be collected. For each test point, the positioning result based on LOS BSs and GDOP of LOS BSs are calculated. Then, for each NLOS BS, estimate new positioning results based on the NLOS BS and all LOS BSs, and calculate a new GDOP. Afterwards, calculate the positioning error change and GDOP change caused by the introduction of the NLOS BS. After all the test points, a data set of two-dimensional arrays, that composed of positioning error change and corresponding GDOP changes, could be obtained. Using the data set, it is easy to find the best threshold, λ, with statistical learning method. For example, before the simulation in Section 5.3, the data set of positioning error changes and GDOP decrement rate is collected, as shown in Figure 4a. Then, use a linear regression with a function, $f\left(x\right)=a_{1}+a_{2}x+a_{3}x^{2}+a_{4}x^{3}$, to fit the data [32]. Using least squares method, it is easy to calculate that $a_{1}=-2.971,\;a_{2}=29.05,\;a_{3}=-96.884,\;a_{4}=80.684$, and the zero of function is about 0.818, as shown in Figure 4b. Therefore, for the simulation in Section 5.2, threshold λ could be set as 0.818.

## 5. Simulation Results and Analysis

### 5.1. Simulation Scenario

**Figure 5:**
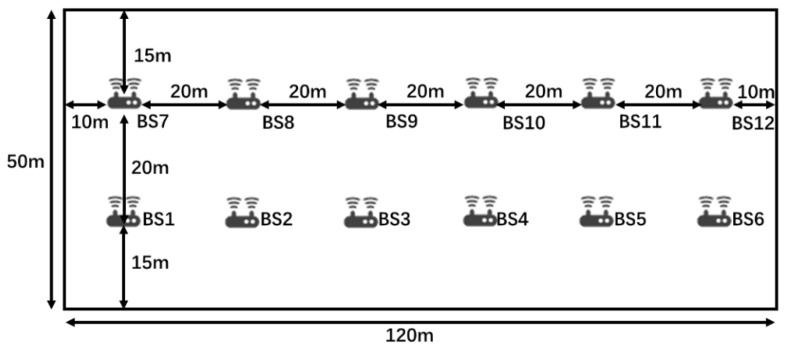
Simulation Scenario with 12 base stations (BSs).

To analyze the GDOP for the hybrid TDOA/RTT/DOA positioning under different BS combinations and to verify the effectiveness of the proposed GDOP-assisted BS selection method, we have built a simulation scenario according to the 3GPP TR 38.901 indoor office scenario [33], as shown in Figure 5. In the scenario, all BSs are 3 m high and UE is located at a level of 1 m high.

### 5.2. GDOP Analysis for The Hybrid Positioning

**Figure 6:**
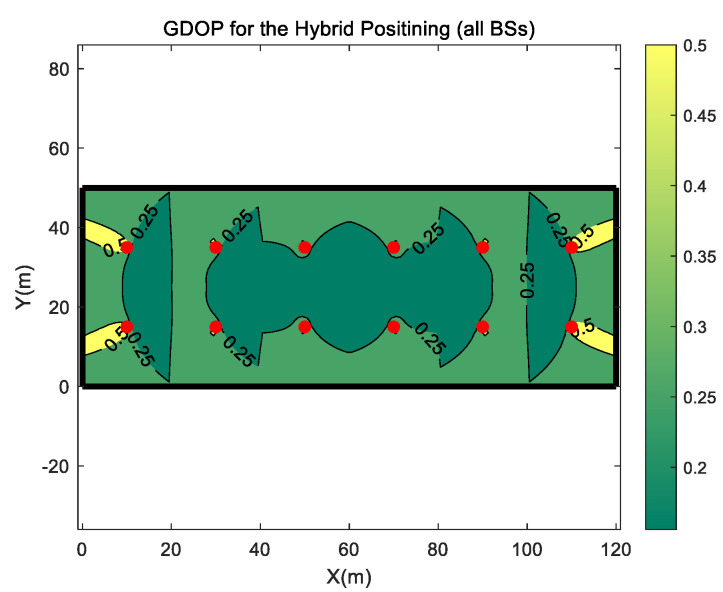
GDOP of the hybrid positioning when all BSs are used for positioning. The red dots indicate BSs.

At first, the GDOP distribution of the hybrid TDOA, RTT and DOA positioning is calculated and shown in Figure 6. According to the measured result in [13], the mm-wave MIMO based 5G system could support a signal arrival time estimation accuracy about $10^{-0.2}\;\approx \;0.631$ ns, which is about 0.189 m, and DOA estimation accuracy about $10^{-3.6}\approx 0.00025$ radian in LOS condition. Therefore, in GDOP calculating, the TDOA measurement error’s standard deviation $\sigma_{tdoa}$ is 0.267 m, the RTT measurement error’s standard deviation $\sigma_{t}$ is 0.189 m, and DOA measurement error’s standard deviation $\sigma_{a}$ is assumed as 0.00025 radian.

Figure 6 shows the GDOP distribution when all BSs are used for positioning. It can be found that most of the area surrounded by the BS has a GDOP value of less than 0.25. Outside the area surrounded by the BSs, the four corner areas of the simulation scene have a little high GDOP value, with values higher than 0.5.

**Figure 7:**
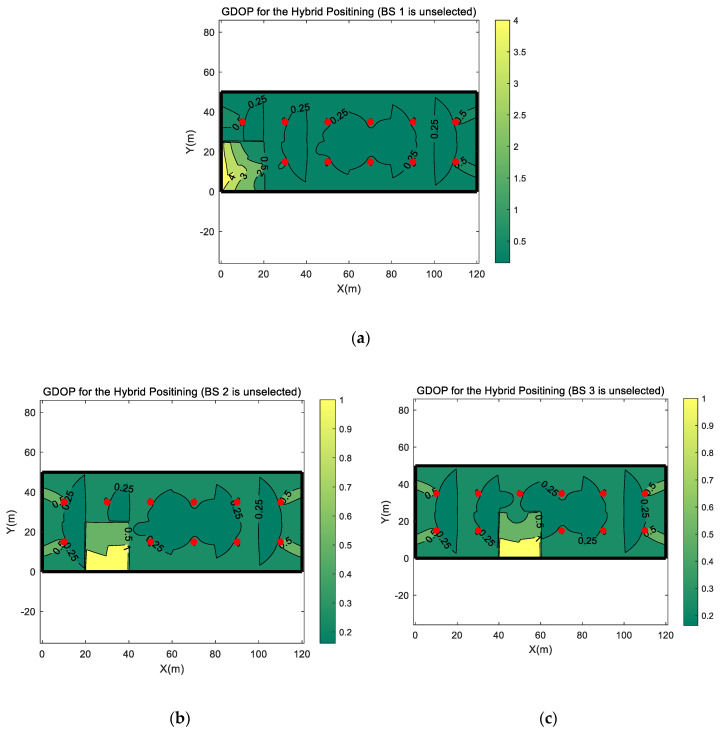
The GDOP distribution of the hybrid positioning when one of the BSs is not selected for positioning. (**a**) The GDOP distribution when BS 1 is not selected. (**b**) The GDOP distribution when BS 2 is not selected. (**c**) The GDOP distribution when BS 3 is not selected.

Figure 7 shows the GDOP distribution when one of the BSs is not selected for positioning. As the used simulation scenario which is recommended by the 3GPP is symmetrical, we only show the GDOP distribution when BS 1, BS 2, or BS 3 is not selected for positioning in Figure 7. The GDOP distribution when another BS is not selected can be obtained by rotating or flipping these three figures. It can be seen from the three figures in Figure 7 that the absence of a BS in positioning can cause an impact on the GDOP value of every locations in the whole simulation scenario. But the increase in the GDOP value outside the service area of the unselected BS is relatively small, while the increase within the service area is much higher. The reason is that, when the serving BS is not used for positioning, the RTT, DOA and a TDOA measurement, provided by the serving BS, are absent in the positioning process. Only the TDOAs from other non-serving BSs could be used for positioning. The system estimates UE position based on TDOA-only but not hybrid TDOA, RTT, and DOA positioning. Therefore, the reduction in number of positioning measurements has a great impact on positioning accuracy. But, in the serving area of other BSs, RTT and DOA measurements are available since the serving BS is still selected for positioning. For the UE positioning in those area, the absence of one non-serving base only causes the loss of one TDOA measurement in positioning and has relatively little effect on GDOP value.

In Figure 7a, it can be found that the GDOP value in BS 1 serving area would increase significantly when BS 1 is not used for positioning. When BS 1 is used for positioning, only a small region in BS 1 serving area has a GDOP higher than 0.5. When BS 1 is not selected, the GDOP value rises to 4, and in most region, it is higher than 2. However, for other two BS selection combination, in Figure 7b,c, the increase in GDOP value, which is caused by the unselected BS 2 or BS 3, is much lower than BS 1, higher than 1 and less than 2, respectively. From those three figures, it can be found that BSs on the two side regions of the simulation scenario have a greater impact on the GDOP value.

**Figure 8:**
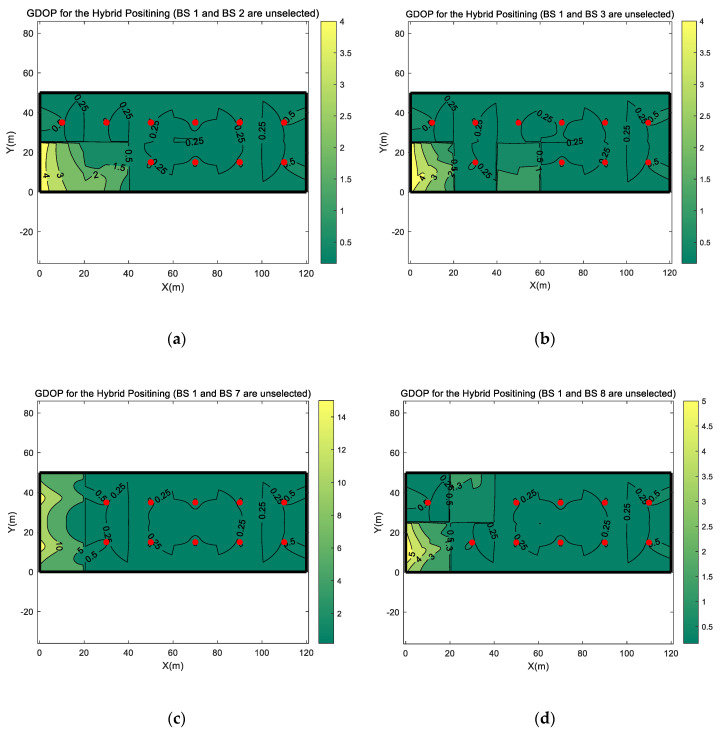
The GDOP distribution of the hybrid positioning when BS 1 and another BS are not selected for positioning. (**a**) the GDOP distribution when BS 1 and BS 2 are not selected; (**b**) the GDOP distribution when BS 1 and BS 3 are not selected; (**c**) the GDOP distribution when BS 1 and BS 7 are not selected; (**d**) the GDOP distribution when BS 1 and BS 8 are not selected.

After the analysis from Figure 7, based on the absence of BS 1, that has a greater impact on GDOP, we further analyze the impact on the GDOP value when another BS is also not selected for positioning. The calculated GDOP distributions are shown in Figure 8. Comparing Figure 7a and Figure 8, it can be found that the absence of another BS does not cause an obviously GDOP changes in selected BSs’ serving area. However, under different BS selection combinations in Figure 8, the change of the GDOP value in the serving area of the unselected BS is different.

In the comparison of the four different BS selection combinations in Figure 8, when the two BSs are not selected in the same side region of simulation scenario, the GDOP value increases more. In Figure 8a,b,d, when only one unselected BS is located at side region, although the GDOP value has increased slightly, the change is much smaller than that in Figure 8c in the serving areas of two unselected BSs. Comparing with Figure 7a, for the three BS selection combination, the GDOP increments in the BS1 serving area are change slightly, the GDOP value at the same location has been increased by up to about 1 with the largest change as shown in Figure 8d. Comparing with Figure 7b,c, the GDOP increment in the other unselected BS’s serving area also changes, but as shown in the BS 2 serving area in Figure 7b and Figure 8a, the GDOP has only increased by about 1. Oppositely, as shown in Figure 8c, when both two unselected BSs are located at side regions of the simulation scenario, the increase in GDOP value is incredibly significant. In Figure 8c, the GDOP value can be as high as 15. In Figure 6, in the same area, when all BSs are selected for positioning, the GDOP value is only 0.5. With two different BS selection combinations, the ratio of GDOP values is as much as 30 times higher.

**Figure 9:**
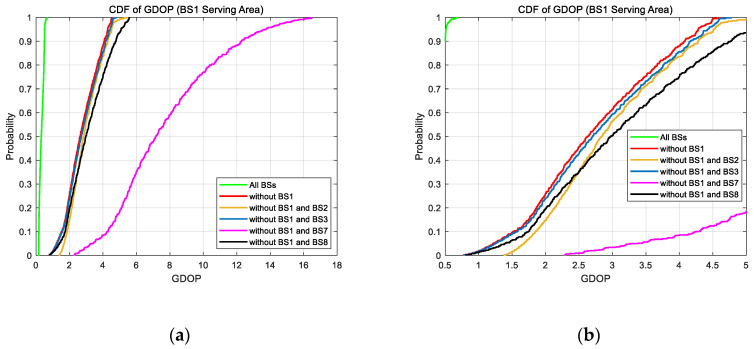
The cumulative distribution functions (CDFs) of GDOP value in BS 1 serving area with different BS selection combinations. (**a**) The overall CDF curves; (**b**) enlarged view of CDF curves when GDOP is 0.5 to 5 in (**a**).

According to the above analyses, to more intuitively show the change of the GDOP value in the side area of the simulation scene, we counted the GDOP value in the BS 1 serving area with different BS selection combinations, which are shown in Figure 6, Figure 7 and Figure 8, and drew their cumulative distribution function (CDF) curves in Figure 9.

As shown in Figure 9, it can be clearly seen that in the areas on the side of the simulation scenario, when the serving BS is not selected for positioning, the GDOP value in this area increases overall. When the two BSs on the same side of the simulation scenario are not selected, the GDOP value in this area increased dramatically.

Based on the above analysis about GDOPs, the different BS selection combinations for positioning could cause a great difference in positioning accuracy. It can be seen from this that if the channel condition-based BS selection method is used, it would greatly increase the GDOP value, which can be several tens of times at the maximum, when the serving BS is in the NLOS condition. According to the definition of GDOP, the increase in GDOP value represents the increase in the lower bound of positioning error. Therefore, the change of the GDOP value caused by BS selection is especially important for positioning.

### 5.3. Simulation Result of Positioning Based on the Proposed GDOP-Assisted BS Selection Method

To show the effectiveness of the proposed GDOP-assisted BS selection method, we computed positioning results, which are based on different BS selection methods, in the simulation scenario with 10,000 Monte Carlo simulations. In each Monte Carlo runs, a UE test point is generated at a random position and the TDOA, RTT, and DOA measurements without errors are calculated. Then, for each BS, the channel condition is randomly determined according to the LOS probability for indoor office scenario in the 3GPP TR 38.901 [33]. For each BS, the LOS probability $P_{LOS}$ is calculated as

(26) $$P_{LOS}=\{\begin{array}{c} \;\;1,\;\;\;\;\;\;\;\;\;\;\;\;\;\;\;\;\;\;\;\;\;\;d_{i}^{\prime }\leq 1.2m\;\;\;\;\;\;\;\\ \;\;exp\left(-\frac{d_{i}^{\prime }-1.2}{4.7}\right),\;\;\;\;\;\;\;\;\;1.2m<d_{i}^{\prime }<6.5m\\ \;\;\exp\left(-\frac{d_{i}^{\prime }-6.5}{32.6}\right)\cdot 0.32,\;\;\;\;6.5m\leq d_{i}^{\prime }\;\;\;\;\;\;\; \end{array},$$

where $d_{i}^{'}$ is the horizontal distance from UE to the BS *I* and, as measured in [13], in NLOS condition, the mm-wave MIMO based 5G system could support a signal arrival time estimation accuracy about $10^{0.7}\approx 5.012$ ns, which is about 1.503 m, and DOA estimation accuracy about $10^{-2.8}\approx 0.0016$ radian. The threshold λ, which is calculated in Section 4, is set as 0.818.

**Figure 10:**
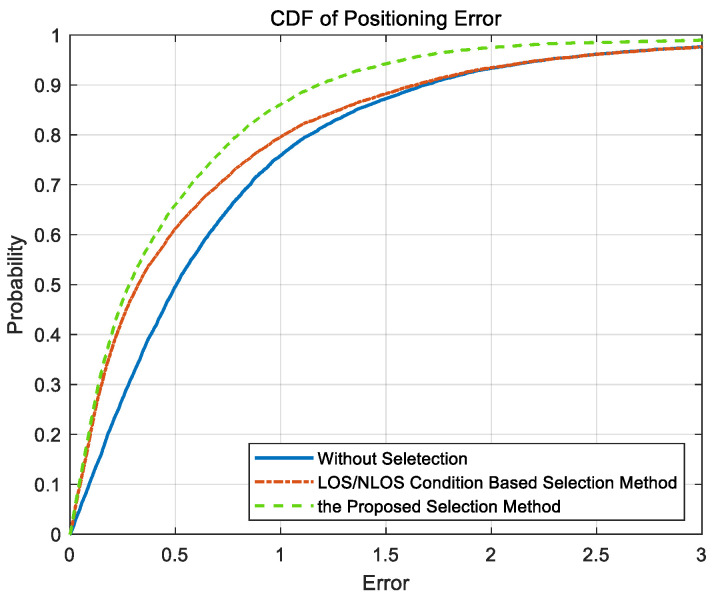
The CDF of positioning error with different BS selection methods.

The cumulative distribution functions (CDFs) of the positioning errors are shown in Figure 10. The yellow curve shows the CDF of positioning errors using the BSs selected by the proposed GDOP-assisted selection method, and the red curve represents the CDF of the positioning errors after the selection of BSs based only on LOS/NLOS channel conditions [20]. The blue curve shows the CDF of the positioning errors calculated based on all BSs. For all three curves, the positioning algorithm in [34] is used. As shown in Figure 10, it is obvious that positioning result based on BSs selected by the proposed method could provide the highest accuracy among the three methods. As existing research has found, the LOS/NLOS condition-based selection method could lead to a higher accuracy than using all BSs without any selection.

**Figure 11:**
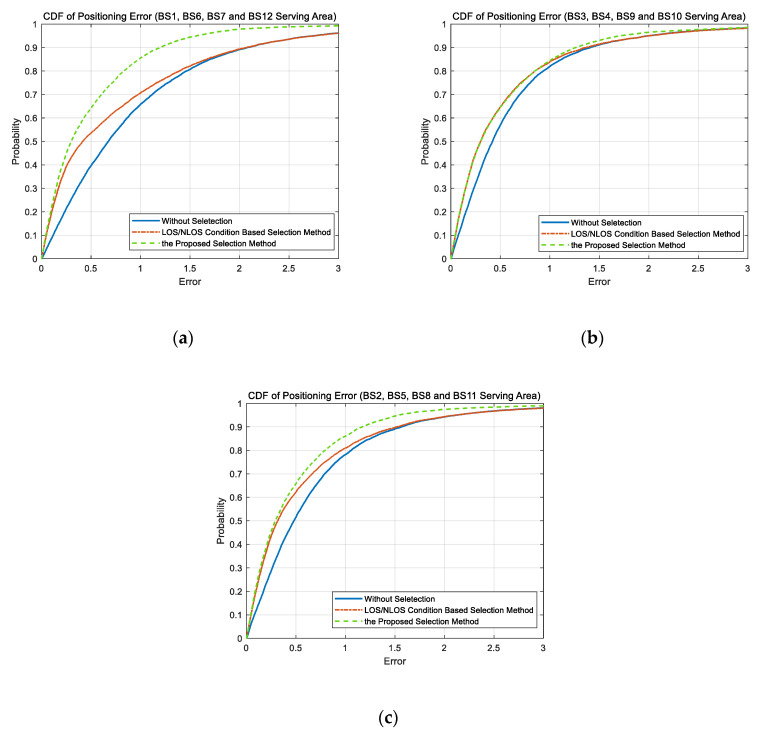
The CDF of positioning error with different BS selection methods in different regions. (**a**) The CDF in the side region. (**b**) The CDF in the center region. (**c**) The CDF in rest of the simulation scenario.

Through the analysis of GDOP in Section 5.2, it can be found that the changes of BS selection combination would lead to a different increment of GDOP in divergent regions of the simulation scenario. To show the effectiveness of the proposed GDOP-assisted BS selection, we also separately counted the positioning errors in different regions. Figure 11a shows the CDF of the positioning errors in two side regions of the simulation scenario, that is, the serving areas of BS 1, BS 6, BS 7, and BS 12. The CDF of the positioning errors in the serving areas of BS 3, BS 4, BS 9, and BS 10, which can be regarded as the center area, is shown in Figure 11b. Comparing the Figure 11a,b, it is obvious that the proposed method is more effective in the side region. In the central area, the effect of the proposed method is close to the LOS/NLOS condition-based BS selection method, and the improvement in positioning accuracy is not obvious. Such results are consistent with the analysis of GDOP in Section 5.2. In the central region, even if the serving BS is not selected for positioning, the increase in GDOP value is small. Therefore, considering only the channel conditions, selecting LOS BSs, which provide a small measurement error, is sufficient to lead to a high positioning accuracy. However, the proposed method is an extension of LOS/NLOS channel condition identify and the channel condition-based selection method, which requires additional calculation steps and has a higher complexity. Therefore, in the center region, the proposed method may be less useful due to the sacrifice in complexity. However, in the side regions, BS selection combination have a great influence on the GDOP value. Although positioning using only the LOS BSs can ensure a high measurement accuracy, it may also cause the GDOP value to increase a lot. Therefore, the proposed GDOP-assisted BS selection method is more effective in these regions. In rest of the simulation scenario, that is, the serving areas of BS 2, BS 5, BS 8, and BS 11, the proposed GDOP-assisted BS selection method is effective and obviously lead to a higher positioning accuracy than LOS/NLOS condition-based BS selection method.

In summary, the proposed GDOP-assisted BS selection method can lead to higher positioning accuracy than the LOS/NLOS condition-based selection method. This improvement is larger in the side region of the simulation scenario and is relatively smaller in the center region.

## 6. Conclusions

In this paper, we propose a GDOP-assisted BS selection method for the hybrid TDOA, RTT and DOA positioning in mixed LOS and NLOS environment. In order to implement the method, we first derived the GDOP for the hybrid positioning. The proposed selection method is an extension of LOS/NLOS channel condition identify and the selection method based only on channel condition [20]. In the proposed method, not only the channel conditions, but also the GDOP increment, caused by the change of BS selection combination used for positioning, is considered. An indoor mm-wave MIMO 5G system scenario is used for simulation and the result shows that the proposed GDOP-assisted BS selection method can lead to higher positioning accuracy than the selection method based only on LOS/NLOS channel condition. In the side region of the simulation scenario, this improvement is more obvious, and it is relatively smaller in the center region. This result is consistent with the theoretical analysis of the GDOP value when different BS selection combination.

## Figures and Tables

**Table 1:** List of symbols.

Symbol	Meaning
$a_{a}$	DOA ranging result
$bs_{i}$	position of BS *i*
$\sigma_{a}^{2}$	Variance of DOA measurement error
$\sigma_{t}^{2}$	Variance of RTT measurement error
$\sigma_{tdoa}^{2}$	Variance of TDOA measurement error
$d_{i}$	Real distance between UE to BS *i*
$d_{i,1}$	Distance difference between $d_{1}$ to $d_{i}$
$d_{1}^{'}$	Real distance from projection of UE to BS *i*
$\vec{e}$	Vector of measurement errors
$e_{a}$	DOA measurement error of signal from UE to BS 1
e	Measurement error, refers to all measurement errors including $e_{a}$, $e_{t}$, and $e_{i}$
$e_{i}$	TDOA measurement error between signals from BS *i* and BS 1 to UE
$e_{t}$	RTT measurement error of UE to BS 1
$e_{x}$	Positioning error on *X*-axis
$e_{y}$	Positioning error on *Y*-axis
$f\left(x^{\prime },y^{\prime }\right)$	Functional relationship between UE positioning result and measurement error, refers to all of $f_{a}\left(x^{\prime },y^{\prime }\right)$, $f_{t}\left(x^{\prime },y^{\prime }\right)$ and $f_{i,1}\left(x^{\prime },y^{\prime }\right)$
$f_{a}\left(x^{\prime },y^{\prime }\right)$	Functional relationship between UE positioning result and DOA measurement error $e_{a}$
$f_{t}\left(x^{\prime },y^{\prime }\right)$	Functional relationship between UE positioning result and RTT measurement error $e_{t}$
$f_{i,1}\left(x^{\prime },y^{\prime }\right)$	Functional relationship between UE positioning result and TDOA measurement error $e_{i}$
$\phi_{1}$	The real azimuth angle between UE to BS 1
$r_{i,1}$	The TDOA ranging result
$r_{t}$	The RTT ranging result
*u*	The position of UE
∆u	The error of UE position estimation result
$\left(x^{0},y^{0}\right)$	UE’s real location
$\left(x^{\prime },y^{\prime }\right)$	Position estimation result
